# Calponin-3 is critical for coordinated contractility of actin stress fibers

**DOI:** 10.1038/s41598-018-35948-6

**Published:** 2018-12-05

**Authors:** Katarzyna Ciuba, William Hawkes, Sari Tojkander, Konstantin Kogan, Ulrike Engel, Thomas Iskratsch, Pekka Lappalainen

**Affiliations:** 1 Insitute of Biotechnology, P.O. Box 56, 0014, University of Helsinki, Helsinki, Finland; 2 School of Engineering and Materials Science, Queen Mary University of London, E1 4NS London, UK; 3 Randall Centre for Cell and Molecular Biophysics, King’s College London, SE1 1UL London, UK; 4 Section of Pathology, Department of Veterinary Biosciences, University of Helsinki, Helsinki, Finland; 5 Nikon Imaging Center at Heidelberg University and Centre for Organismal Studies (COS), Heidelberg University, Im Neuenheimer Feld 267, Heidelberg, 69120 Germany

## Abstract

Contractile actomyosin bundles, stress fibers, contribute to morphogenesis, migration, and mechanosensing of non-muscle cells. In addition to actin and non-muscle myosin II (NMII), stress fibers contain a large array of proteins that control their assembly, turnover, and contractility. Calponin-3 (Cnn3) is an actin-binding protein that associates with stress fibers. However, whether Cnn3 promotes stress fiber assembly, or serves as either a positive or negative regulator of their contractility has remained obscure. Here, we applied U2OS osteosarcoma cells as a model system to study the function of Cnn3. We show that Cnn3 localizes to both NMII-containing contractile ventral stress fibers and transverse arcs, as well as to non-contractile dorsal stress fibers that do not contain NMII. Fluorescence-recovery-after-photobleaching experiments revealed that Cnn3 is a dynamic component of stress fibers. Importantly, CRISPR/Cas9 knockout and RNAi knockdown studies demonstrated that Cnn3 is not essential for stress fiber assembly. However, Cnn3 depletion resulted in increased and uncoordinated contractility of stress fibers that often led to breakage of individual actomyosin bundles within the stress fiber network. Collectively these results provide evidence that Cnn3 is dispensable for the assembly of actomyosin bundles, but that it is required for controlling proper contractility of the stress fiber network.

## Introduction

Contractile actomyosin bundles, stress fibers, are important for morphogenesis, migration and mechanosensing of non-muscle cells. Moreover, stress fibers associate with other cytoskeletal networks, such as cytoplasmic intermediate filaments, and control their subcellular distribution^1–3^. Stress fibers can be divided into different categories, based on their protein composition and interactions with focal adhesions; *Ventral stress fibers* are relatively thick actomyosin bundles that associate with focal adhesions at their both ends. Ventral stress fibers are derived, at least in the U2OS osteosarcoma cells, from a network of dorsal stress fibers and transverse arcs. *Dorsal stress fibers* are non-contractile actin filament bundles that elongate through actin filament assembly at focal adhesion located at their distal end. *Transverse arcs* are relatively thin contractile actomyosin bundles that do not directly associate with focal adhesions^4^.

In addition to actin and non-muscle myosin II (NMII), stress fibers are composed of a large array of actin-associated and signaling proteins. Importantly, the specific functions of many stress fiber-associated proteins have remained elusive, and thus the precise molecular details underlying the assembly and contractility of stress fibers are unknown^4^. One of the stress fiber-associated proteins, which functions are incompletely understood is calponin. It was first identified from chicken gizzard and characterized as a protein that binds F-actin, calmodulin, and tropomyosin^5^. In vertebrates, three calponin-encoding genes (*CNN1*, *CNN2*, and *CNN3*) express three isoforms (known as calponin-1, calponin-2, calponin-3)^6–8^. The three isoforms are approximately 70% identical to each other at the amino acid level and they are all composed of an N-terminal calponin homology (CH) domain, followed by two actin-binding sides, three calponin-like (CLIK) motifs, and a divergent C-terminal tail region. The variable lengths and amino acid compositions of the C-terminal region result in different charge features in this motif, and hence Cnn1 is also known as basic calponin, Cnn2 as neutral calponin, and Cnn3 as acidic calponin^6,7,9^.

The best characterized calponin isoform is Cnn1, which is mainly restricted to smooth muscle cells. A wealth of biochemical and *in vivo* studies provided evidence that Cnn1 controls smooth muscle contractility by inhibiting actin-activated myosin ATPase activity without affecting phosphorylation of the myosin regulatory light chain (MLC)^5,10–12^. Similarly, genetic studies on the *C. elegans* homologues of calponin demonstrated that they function as negative regulators of actomyosin contractility *in vivo*^13^. However, other studies provided evidence that Cnn1 may also affect the mechanical properties of actin filaments and regulate organization of the actin cytoskeleton in smooth muscle cells^14,15^.

Cnn2 and Cnn3 are widely expressed in non-muscle and smooth muscle cells^16–23^. Cnn2 knockout mice are viable, but display defects in the proliferation and migration of macrophages, neutrophils and monocytes^24,25^. In contrary, Cnn3 is essential for embryonic development and viability of mice^23,26^. The precise functions of Cnn2 and Cnn3 in non-muscle cells remain somewhat controversial. Both proteins localize to contractile actomyosin bundles, such as stress fibers^16,21,27^. Similar to Cnn1 in smooth muscle cells, Cnn2 depletion results in increased traction forces, suggesting that this protein is a negative regulator of actomyosin activity^18^. In contrast, knockdown of Cnn3 in primary fibroblasts was reported to reduce contractility, most likely due to impaired stress fiber assembly^27^. Cnn3 was also shown to regulate cell contractility through MEKK-1 signaling pathway in MLC independent manner^28^. Other studies provided evidence that Cnn2 and Cnn3 may stabilize actin filaments, and thus control the organization of the actin cytoskeleton^16,27,29^. Here, we applied the U2OS cell model system to reveal whether Cnn3 primarily controls organization of the actin cytoskeleton, or functions as a regulator of actomyosin contractility. We show that Cnn3 is a dynamic component of stress fibers, and that its depletion results in increased and uncontrolled contractility of the stress fiber network. These results provide evidence that, like Cnn1, also Cnn3 functions as a negative regulator of actomyosin contractility in cells.

## Results

### Calponin-3 is a dynamic component of contractile stress fibers

**Figure 1:**
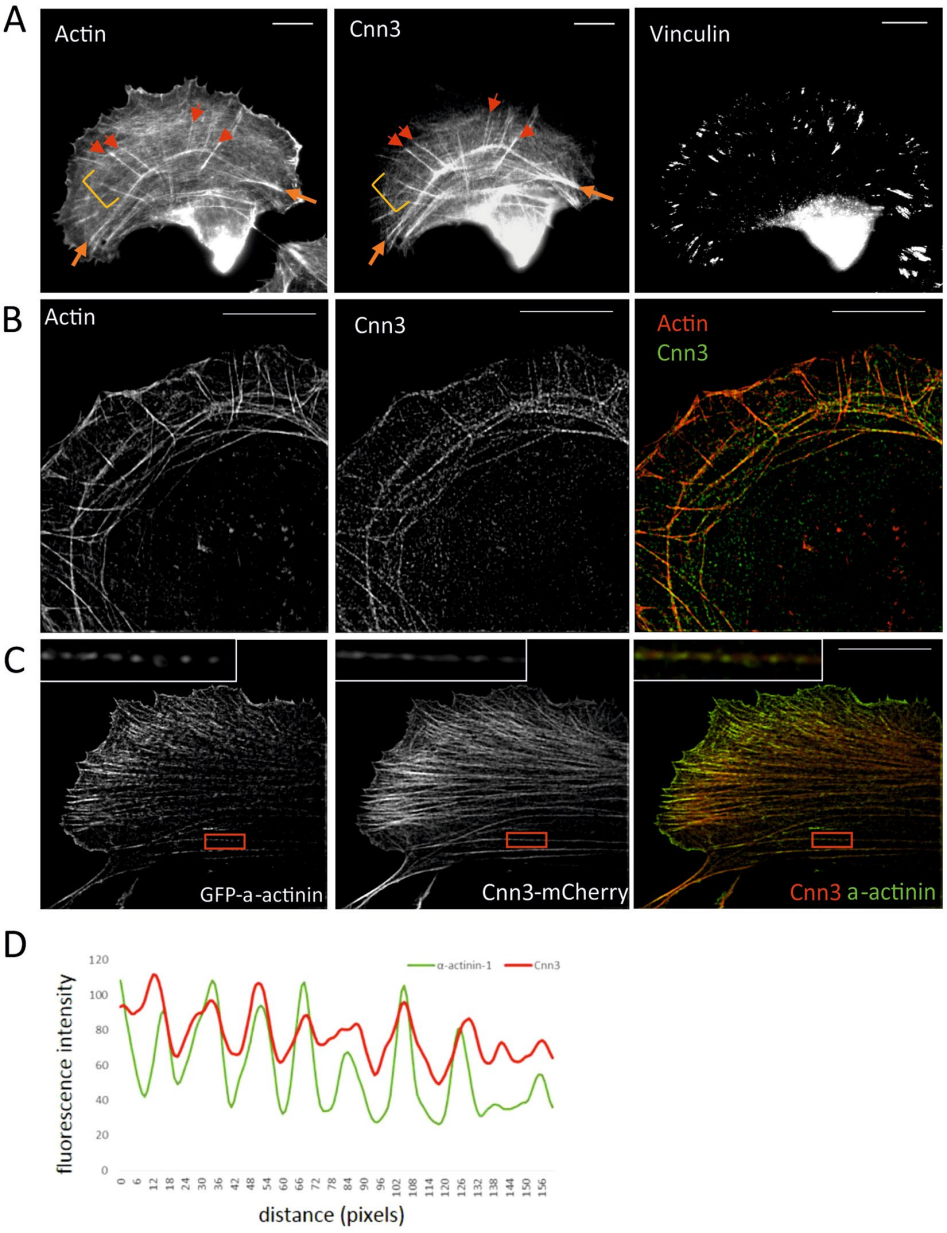
Localization of Calponin-3 in U2OS cells. (**A**) Endogenous Cnn3 localizes to all three stress fibers categories in U2OS cells. Examples of dorsal stress fibers are marked with red arrows, ventral stress fibers with orange arrows, and thin transverse arcs with yellow brackets. (**B**) Localization of endogenous Cnn3 to actin-rich (visualized by fluorescent phalloidin) stress fibers by 3D-SIM. (**C**) Co-localization of Cnn3-mCherry with GFP-α-actinin-1 in stress fibers as detected by 3D-SIM. The red box represents a zoomed region containing a segment of a ventral stress fiber (that is included as an inset in the top-left corner of the images). (**D**) The same region was analyzed with a line profile tool to illustrate the co-localization of Cnn3 to α–actinin-1 –rich regions along the stress fiber. Single slices of 3D-SIM images are presented. Scale bars, 10 µM.

Calponin-3 (Cnn3) localizes to actin stress fibers (Fig. 1A)^1,28,30^, but its precise localization pattern within the stress fiber network has not been reported. Therefore, we applied 3D structural-illumination microscopy (3D-SIM) imaging to study the localization of Cnn3 in human osteosarcoma (U2OS) cells. U2OS cells have been extensively used as a model system for studying the assembly and contractility of stress fibers (e.g.^31–35^). The conventional immunofluorescence microscopy and 3D-SIM experiments demonstrated that both endogenous Cnn3 (Fig. 1A,B) and exogenously expressed Cnn3-mCherry (Fig. 1C) localize to all three stress fibers categories; dorsal stress fibers, transverse arcs, and ventral stress fibers. 3D-SIM experiments further revealed that in U2OS cells Cnn3 is not uniformly distributed along stress fibers, but it is enriched in α-actinin-1 foci in transverse arcs and ventral stress fibers (Fig. 1C,D). Consistent with these data, Cnn3 is also present in non-contractile dorsal stress fibers that are enriched with α-actinin-1, but do no not contain myosin II (Suppl. Fig. 1A).

**Figure 2:**
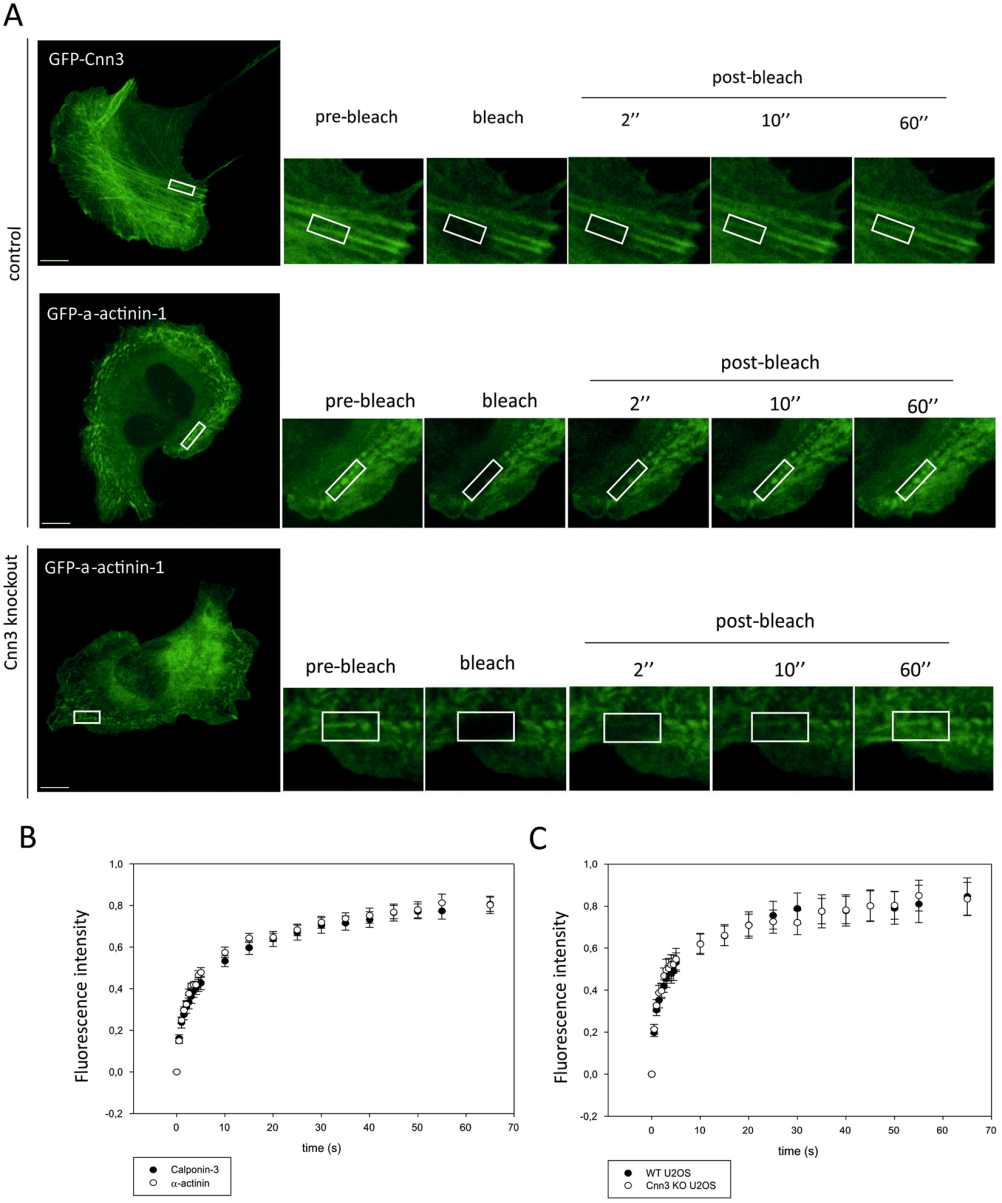
Calponin-3 is a dynamic component of contractile stress fibers. (**A**) Cnn3 and α-actinin-1 dynamics were measured by FRAP in contractile fibers. Representative GFP-α-actinin-1 and GFP-Cnn3 expressing cells used in the experiments are shown of the left, and fluorescence recoveries of each case shown on the right. White boxes represent regions of interest (ROI). Scale bars, 10 μm. (**B**) Plots representing the rates of fluorescence recovery of Cnn3 and α-actinin-1 in stress fibers after photobleaching in wild-type U2OS cells. (**C**) The rates of α-actinin-1 fluorescence recovery in stress fibers of wild-type and Cnn3-depleted U2OS cells. n = 10 cells for all experiments. The data are presented as +/− SEM.

Actin-binding proteins display large differences in the dynamics of their stress fiber associations. For example, VASP and palladin display highly dynamic (half-time ~1 s) association with stress fibers, whereas α-actinin-1 displays intermediate dynamics (half-time ~2–5 s), and tropomyosins exhibit more stable (half-time ~5–10 s) association with stress fibers^32,36^. To reveal the dynamics of Cnn3 in stress fibers, we performed fluorescence-recovery-after-photobleaching (FRAP) experiments on cells expressing GFP-Cnn3, and monitored the recovery of GFP-Cnn3 fluorescence on mature, contractile ventral stress fibers. This analysis revealed that Cnn3 exhibits a half-life of approximately ~2–4 s on stress fibers (Fig. 2A,B), and hence displays similar turnover on stress fibers with α-actinin-1 (Fig. 2B). Together these data demonstrate that Cnn3 is a dynamic stress fiber component, which co-localizes with α-actinin-1 in all three stress fiber categories of U2OS cells.

### Calponin-3 is dispensable for stress fiber assembly

Previous RNAi studies suggested that Cnn3 is important for the assembly of stress fibers in dermal fibroblasts^27^. To examine the possible role of Cnn3 in stress fiber assembly in osteosarcoma cells, we generated Cnn3 knockout U2OS cells by CRISPR/Cas9. Sanger sequencing of the obtained Cnn3 knockout cell clone revealed disruption of the genomic region of *CNN3* (Suppl. Fig. 2A). Moreover, MiSeq next generation sequencing of genomic DNA from *CNN3* knockout cells confirmed that they were unable to synthetize functional Cnn3. We found three distinct CRISPR/Cas9-induced effects, all altering the reading frame, either by deletion of 4 nucleotides, or insertion of 1 and/or 2 nucleotides (Suppl. Fig. 2B). This indicates that U2OS osteosarcoma cells have three copies of *CNN3* gene, most likely due to aneuploidy. Each variant in the Cnn3 knockout cell-line resulted in premature termination of translation, as stop codons were introduced in the exon 2 (Suppl. Fig. 2C). Finally, complete absence of Cnn3 protein was confirmed from the knockout cells by Western blotting using Cnn3-specific antibody (Suppl. Fig. 3B).

**Figure 3:**
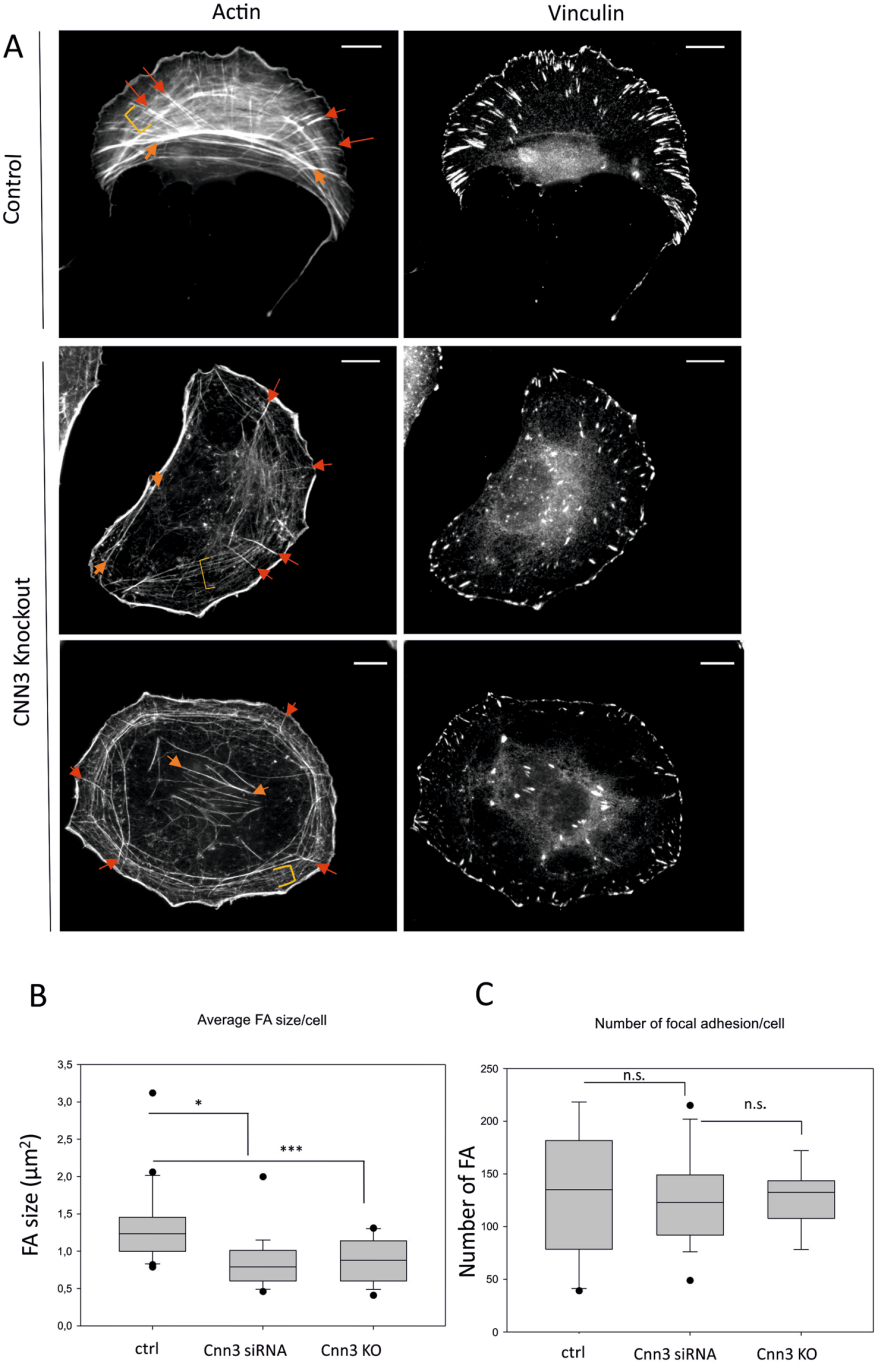
Calponin-3 is not critical for stress fiber assembly. (**A**) Actin filaments (fluorescent phalloidin) and focal adhesions (anti-vinculin) were visualized in control and Cnn3 knockout cells. The three categories of stress fibers are indicated with arrows and brackets as in Fig. 1A. Scale bars, 10 µM. (**B**,**C**) Average areas of focal adhesions (panel B) and numbers of focal adhesions per cell (panel C) were quantified from control as well as Cnn3 knockout and knockdown cells. n (cells): control (20), Cnn knockdown (19), Cnn knockout (16). Box charts have Whisker range 5–95, showing outliers. Statistical significance was assessed with Mann-Whitney test; n.s. (not-significant), p < 0.005 (*), p < 0.001 (***).

By visualizing actin filaments from control and Cnn3 knockout cells by phalloidin staining, we revealed that Cnn3 knockout U2OS cells were still able to generate all three categories of stress fibers (Fig. 3A). However, in many cells the morphology of the stress fiber network was altered. This is because actin bundles were often thinner and less prominent compared to the control cells, and the arrangement of stress fiber networks in Cnn3 knockout cells was somewhat less regular compared to the control cells. However, α-actinin-1, which co-localizes with Cnn3 in stress fibers and displays very similar dynamics with Cnn3 within the stress fiber network, still accumulated to stress fibers in Cnn3 knockout cells and displayed indistinguishable dynamics compared to the control cells (Fig. 2A,C; Suppl. Fig. 1B,C). Thus, the defects in the organization of the stress fiber network in Cnn3 knockout cells do not arise from altered localization or dynamics of α-actinin-1 in the absence of Cnn3.

We also applied vinculin staining to uncover the possible effects of Cnn3 depletion on focal adhesions. By quantifying the numbers and sizes of focal adhesions in control and Cnn3 knockout cells, we revealed that the average number of focal adhesions was very similar in control and Cnn3 knockout cells, but that the average areas of focal adhesions in Cnn3 knockout cells were significantly smaller compared to the control cells (Fig. 3B). To confirm that the observed phenotypes do not result from CRISPR/Cas9 off-target effects, we also silenced Cnn3 from U2OS cells by RNAi (Suppl. Fig. 3A,B). Phalloidin staining and focal adhesion analysis revealed very similar effects of Cnn3 depletion by siRNA on the organization of the stress fiber network and average sizes of focal adhesions and number compared to the Cnn3 knockout cells (Fig. 3B,C; Suppl. Fig. 3C). Thus, Cnn3 is dispensable for stress fiber assembly, at least in U2OS cells.

### Loss of calponin-3 leads to uncontrolled contractility of the stress fiber network

**Figure 4:**
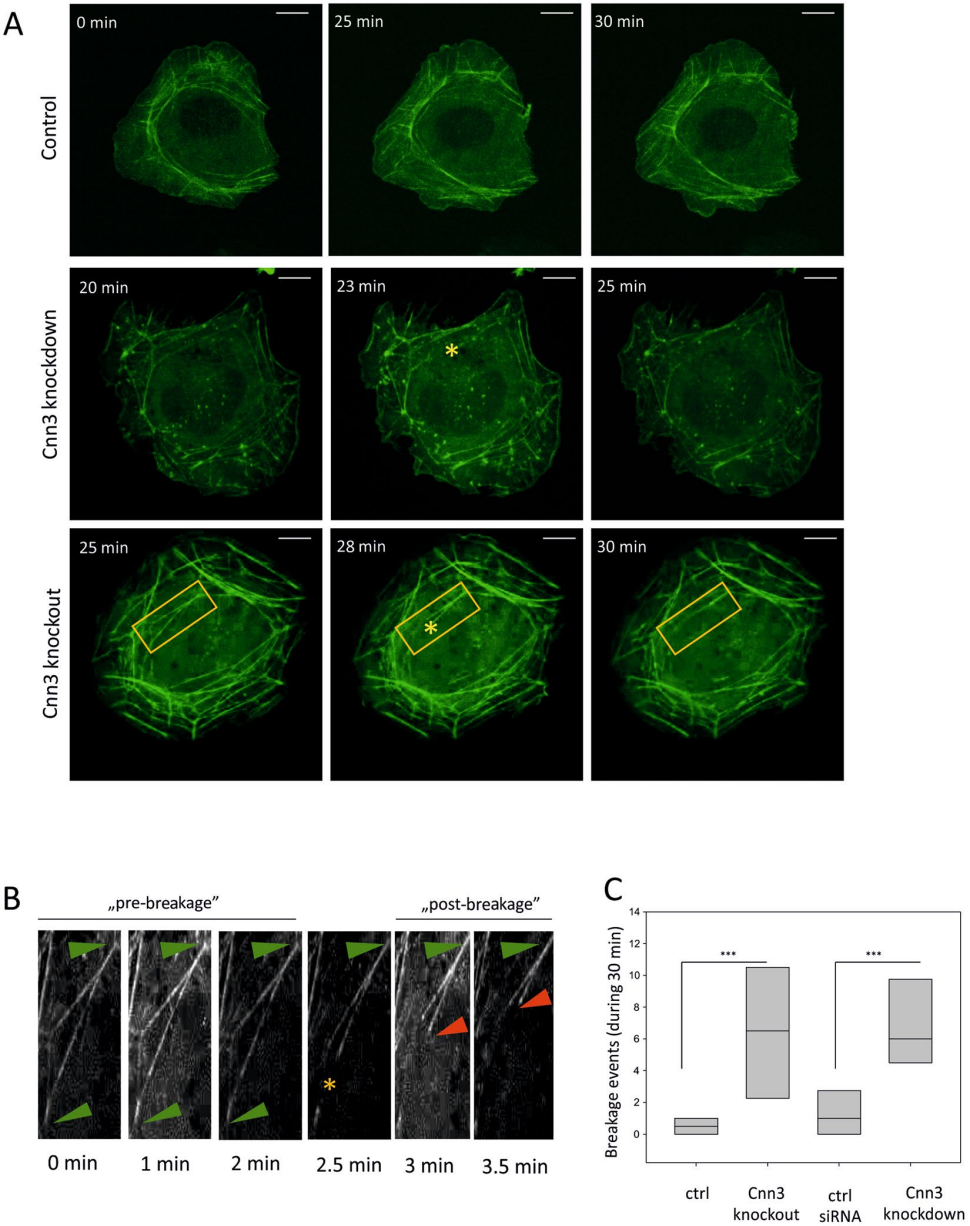
Loss of Calponin-3 impairs stress fiber contractility. (**A**) Control, Cnn3 knockdown, and Cnn3 knockout U2OS cells transfected with GFP-actin were imaged for 30 min, and frames were captured every 30 s. Representative time-lapse frames for each condition are presented. Examples of stress fiber breakage events in Cnn3 knockdown and knockout cells are marked by yellow asterisks (‘*’). Scale bars, 10 μm. Please, see supplemental videos 1–3 for better visualization of abnormal stress fiber dynamics and breakage events. (**B**) Time-lapse frames from the region indicated by an orange box in panel ‘A’ representing a detailed view of stress fiber breakage event. Green arrowheads show the ends of this individual actomyosin bundle, yellow star the breakage point, and red arrowhead one end of the retracting fiber after the breakage. (**C**) Numbers of breakage events in each cell type was quantified in 8 cells per each condition. Statistical significance was assessed with Mann-Whitney test; p < 0.001 (***).

Although cells lacking Cnn3 were able to generate stress fibers, the stress fiber networks in Cnn3 knockout and knockdown cells typically displayed abnormalities in their organizations. To gain insight into these stress fiber network organization defects, we performed live-imaging of control and Cnn3-deficient (both knockout and knockdown) U2OS cells, transiently transfected with a plasmid expressing GFP-actin. Importantly, these experiments revealed major disruption of stress fiber network contractility in Cnn3-deficient cells. Compared to control cells, which displayed ‘smooth’ retrograde flow of transverse arcs and balanced contractility of ventral stress fibers (Supplemental Movie 1), the Cnn3 knockout and knockdown cells typically exhibited abnormal, disorientated *tug-of-war* behavior of stress fibers (Supplemental Movies 2 and 3). This uncoordinated contractility was often accompanied by breakage of the stress fibers (Fig. 4A,B). By quantifying the numbers of stress fiber breakage events in control as well as Cnn3 knockdown and knockout cells from 30 min live-cell movies, we revealed that frequency of stress fiber breakage events was increased >10-fold in Cnn3 deficient cells compared to the control cells (Fig. 4C).

**Figure 5:**
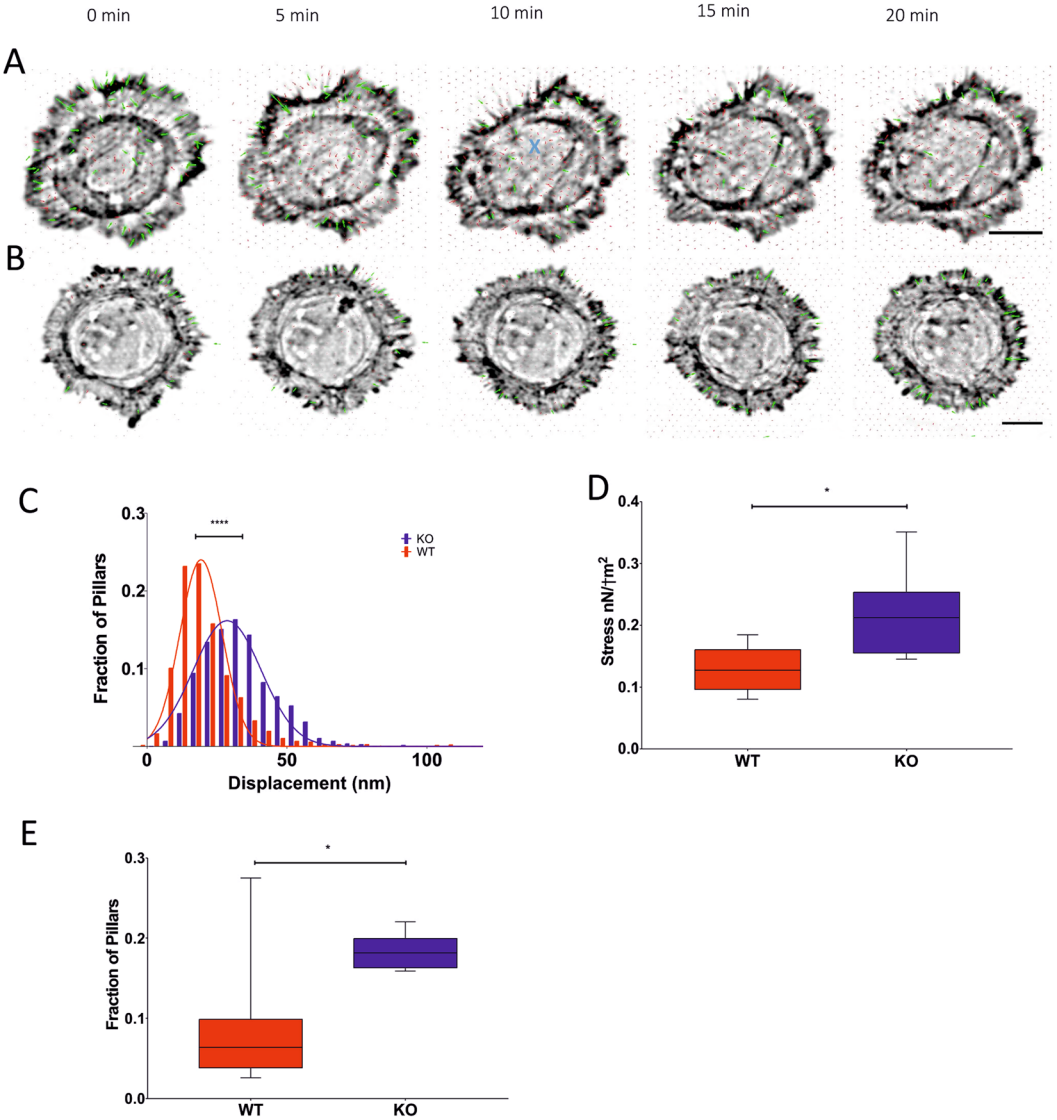
Cnn3-deficient cells exhibit increased traction forces. Composite images of live-imaging of Cnn3-knockout (**A**) and control (**B**) cells; the Tractin channel (black and white) and pillar traction forces (arrows). Green and red arrows indicate displacements above or below 30 nm, respectively. The blue X at 10 minutes indicates the time and site of stress fiber breakage in the example Cnn3-knockout cell. Scale bars, 10 μm. (**C**) Pillar displacements, (**D**) traction forces and (**E**) fraction of pillars displaced underneath each cell were quantified. n > 1,000 pillars per condition from 6 WT and 8 CNN3-KO cells. Bars = min – max. Student’s t-test: p < 0.05 (*); p < 0.0001 (***).

We next applied pillar-based traction force measurements combined with live cell imaging to elucidate whether the abnormal behavior and breakage of stress fibers in Cnn3 knockout cells results from increased/unregulated contractility of stress fibers (Fig. 5A,B). Pillar displacements were significantly greater in Cnn3 knockout cells compared to control cells, resulting in a larger traction stress per cell (Fig. 5C–E), which was due to a larger fraction of pillars under the cell that showed displacements above the noise level (30 nm). Breakage events were also observable on nanopillars. Because of the breakages occurring in a network of interlinked stress fibers, it was not possible to track the forces back to individual adhesions on pillars. Nevertheless, we frequently observed a buildup of traction forces prior to the breakage and a drop of forces on individual pillars close to the breakage point after the event (Suppl. Fig. 4). Therefore the results of the pillar experiments further underline the hypothesis that the breakages occur due to uncoordinated contractility, possibly enhanced through a structural instability of the thinner stress fibers in the Cnn3 knockout cells.

## Discussion

Several studies have shown that Cnn1 and Cnn2 function as negative regulators of actomyosin ATPase activity *in vitro* and myosin II -driven contractility in cells (e.g.^10,18,37^). In contrast, the ubiquitously expressed Cnn3 was proposed to regulate the assembly of actin stress fibers in fibroblasts, and therefore have a positive impact on myosin II-driven contractility of non-muscle cells^27^. Here, we examined the role of Cnn3 in the assembly and contractility of stress fibers in U2OS cells, which have been extensively used as a model system for determining the cellular functions of cytoskeletal proteins.

Several actin-binding proteins display specific localization patterns within the stress fiber network. For example, palladin and vasodilator stimulated phosphoprotein (VASP) are enriched in non-contractile dorsal stress fibers, whereas non-muscle myosin II isoforms and certain tropomyosins (e.g. Tpm4.2) localize only to transverse arcs and ventral stress fibers, but are absent in dorsal stress fibers^32,36^. Importantly, our data revealed that Cnn3 is present in all three categories of stress fibers, where it co-localizes with α-actinin-1. Moreover, Cnn3 displays similar rapid dynamics on stress fibers as α-actinin-1. However, it does not appear to function as a regulator of α-actinin-1, because Cnn3 depletion did not affect the subcellular localization of α-actinin-1 or its dynamics on stress fibers.

Recent RNAi studies provided evidence that Cnn3 is critical for stress fiber assembly in dermal fibroblasts^27^. However, our CRISPR/Cas9 knockout and RNAi knockdown studies revealed that at least in U2OS cells Cnn3 is not required for stress fiber assembly, as all three categories of stress fibers were still present in Cnn3-depleted cells. Nevertheless, the morphology and especially the dynamic behavior of the stress fiber networks in Cnn3-deficient cells was abnormal. This is particularly evident from live-cell imaging experiments, which revealed peculiar *tug-of-war* behavior of stress fibers in Cnn3-depleted cells (Supplemental Movies 2 and 3), as well as increased physical breakage of individual stress fibers with the network (Fig. 4B,C). Traction-force microscopy experiments demonstrated that many stress fibers in Cnn3 knockout cells displayed higher contractility compared to the stress fibers of control cells, and that these increased tractions led to breakage of stress fibers. Although the contractility of stress fibers in Cnn3-depleted cells was increased, the average focal adhesions areas were smaller. In this context it is important to note that, although increased traction forces are linked to enlargement of focal adhesions during their assembly, it was reported that tension and adhesion size do not strictly correlate in mature focal adhesions^38^. Therefore, the effects of Cnn3 depletion on the average focal adhesion areas may result from differences in maturation stages of the adhesions or from indirect effects of Cnn3 depletion on the composition of focal adhesions. Together, these data demonstrate that Cnn3 functions as an indirect negative regulator of myosin II-driven contractility in U2OS cells.

What is the molecular mechanism by which Cnn3 controls the contractility of stress fibers? As Cnn3 localizes to α-actinin-1 -rich *z-line* regions of stress fibers, it most likely does not directly form a complex with non-muscle myosin II to control its activity. Thus, we propose that Cnn3 may restrict the movement of non-muscle myosin II molecules along the actin filament in stress fibers. Alternatively, Cnn3 may affect the organization or molecular composition of stress fibers in the way that absence of Cnn3 would lead to de-regulated activity of myosin II or its motility along actin filaments within stress fibers. The latter option is supported by the fact that Cnn3 also localizes to dorsal stress fibers that do not contain myosin II.

Cnn3 was earlier shown to form a complex with ERK1/2 and PKCα kinases on stress fibers. In this case, the ERK1/2-Cnn3-PKCα complex would be released from stress fibers upon a stimuli, translocated to the plasma membrane where MEK activates ERK1/2, which subsequently moves back to actin filaments to phosphorylate caldesmon and consequently increase the myosin II-driven contractility^21^. Although this scenario may be important upon specific stimuli in cells, it is unlikely the mechanism by which Cnn3 affects stress fiber contractility in U2OS cells. This is because Cnn3 displays constant, rapid turnover (t_1/2_ = 2–4 s) in stress fibers. Thus, it is difficult to imagine how the fast association/dissociation kinetics of Cnn3 at stress fibers could be linked to such multi-step translocation/phosphorylation pathway, which is expected to be considerably slower^39^. Therefore, although the activity of Cnn3 can be regulated by various kinase pathways in cells^21,28^, we propose that Cnn3 regulates stress fiber organization and contractility in a more direct manner (e.g. by controlling the organization of actin filaments within stress fibers or by regulating the activities or localization of other stress fiber components).

Collectively, our work reveals important new aspects on the cellular function of Cnn3. We show that (1). Cnn3 is a dynamic component of stress fibers, (2). Cnn3 co-localizes with α-actinin-1 in all categories of stress fibers, including the non-contractile dorsal stress fibers, (3). Cnn3 is not required for stress fiber assembly, and (4). Lack of Cnn3 leads to uncontrolled contractility of the stress fiber network. In the future, it will be important to examine the precise molecular function of Cnn3 in stress fiber contractility and organization in the context of its reported interaction partners, for example tropomyosins.

## Methods

### Cell culture and treatments

Human osteosarcoma (U2OS) cells were cultured in DMEM with 10% BSA and 1% Penicilin/Streptomycin, in an incubator (+37 C °C, 5% CO_2_). Following plasmids were used in this study: GFP-Cnn3 (gift from M. Gimona^30^); Cnn3-mCherry (Cnn3-encoding fragment was PCR-amplified with 5′-TATCTCGAGCTATGAACAAGCTGCAGCC, 5′-TATGAATTCGATCAGTAGCCGGCTTCCTC primers and subcloned into pmCherry-C1 vector into XhoI/EcoRI sites); GFP-actin and GFP-α-actinin-1^31^. Cells were transiently transfected with FuGENE HD transfection reagent (Promega), according to the manufacturer’s instructions. Cells were then incubated for 24 h and either fixed with 4% PFA or re-plated on fibronectin-coated, glass-bottomed dishes for live cell imaging. For Cnn3-knockdown *ON-TARGETplus Smartpool* L-011612-02-0005 (Dharmacon) was used, and *AllStars negative control siRNA* (Qiagen) was used for control cells. Cells were incubated for 72–96 h for efficient depletion of the target protein.

### Cell Imaging

All immunostainings (for wide-field and 3D-SIM) were performed as described previously^1^. The following antibodies were used for immunofluorescence stainings: mouse 1:100 anti-Cnn3 (A-2, SCBT); 1:400 rabbit anti-Vinculin (ab73412, Abcam); 1:200 rabbit anti-NMIIA (909801, BioLegend). F-actin was visualized with 1:200 Alexa Fluor 488-, 568-, or 647-phalloidin (Invitrogen). Secondary anti-mouse or anti-rabbit antibodies were conjugated to Alexa Fluor488-, 568-, or −647 (Invitrogen). Wide-field images were taken with LeicaDM6000 upright fluorescence wide field microscope (63x/1.40 − 0.60 HCX PL APO Oil objective), equipped with Hamamatsu Orca-Flash4.0 V2 sCMOS camera. 3D-SIM images were taken with Nikon N-SIM structured illumination microscope on a Nikon Ti inverted microscope (Nikon Apo TIRF 100x NA 1.49 oil immersion objective), equipped with Andor iXon3 DU-897E single photon detection EMCCD camera (as described in^40^). For live-imaging and FRAP, transfected cells were re-plated prior to imaging on fibronectin-coated (10 μg/ml), glass-bottomed dishes. The time-lapse images were acquired in heated sample chamber (+37 °C, CO_2_). 3I Marianas imaging system (3I Intelligent Imaging Innovations), with an inverted spinning disc confocal microscope Zeiss Axio Observer Z1 (63x/1.2 W C-Apochromat Corr WD = 0.28 M27 objective) and a Yokogawa CSU-X1 M1 confocal scanner were used. Slidebook 6.0 software and sCMOS (Andor) Neo camera were used for the image acquirement and recording. Image analysis were done with ImageJ.

### Western Blot

Cells were washed with ice-cold PBS, then lysed with 4xLSB-DTT. Lysates were briefly sonicated prior to boiling and loaded on SDS-PAGE gels. Trans-Blot turbo system (BioRad) was used for transfer. After 1 hour blocking (5% BSA), following antibodies were used for detection of proteins: 1:1000 mouse anti-Cnn3 (A-2, SCBT), 1:5000 mouse anti-tubulin (T9026, Sigma-Aldrich). Anti-mouse HRP secondary antibody (Invitrogen) and Western Bright ECL-spray (Advansta) were used for chemiluminescence detection of the protein bands.

### CRISPR/Cas9 knockout

GFP-Cnn3 CRISPR/Cas9 construct and knockout cell line were generated as previously described^41^, with target sgRNA sequence: TCAACAAGGGCCCTTCCTAT. knockout was confirmed with Western Blot, and by sequencing of the genomic DNA. For sequencing, genomic DNA was isolated from wild-type and Cnn3 knockout U2OS cells, the sgRNA targeted area was amplified with 5′-CTCTGTAGCACCCAGTTGGA and 5′-TTCTGCAGTCACCAAACGG primers and sent to Sanger sequencing reaction first. Due to non-interpretable overlap of the multiple signals we also performed MiSeq next generation sequencing of the same locus. The total of around 200 000 reads were analyzed from different PCR products by Geneious software.

### Pillars Assay

PDMS nanopillars (d = 0.5μm, h = 1.7 μm, bending stiffness k = 3pN/nm) were fabricated and coated with CdSeS/ZnS alloyed quantum dots (490 nm, Sigma) and fibronectin as described previously^42^. Tractin-tdTomato transfected WT and CNN3-KO U2OS cells were plated for 1 hour on PDMS nano-pillar arrays and imaged every 30 seconds for ≥20 minutes on a Nikon Eclipse Ti Inverted spinning disk confocal with a Yokogawa CSU-1 disk head, Andor Neo sCMOS camera and equipped with a Solent Scientific chamber with temperature and CO2 control. After imaging cells were trypsinized to recover the original pillar positions. Pillar displacements were analysed with imageJ, using the PillarTracker plugin. Final deformations and forces were calculated with Matlab and statistics and graphs were generated with Graphpad Prism 7.

### Statistical analyses

Statistical analyses and all charts, except pillar assay analysis, were done with Sigma Plot 11.0 software. Statistical differences in focal adhesion and stress fiber breakage analysis are assessed by the Mann–Whitney–Wilcoxon rank-sum test (MWW), and in pillar assay analysis by the Student t-test.

The datasets generated and analyzed during the current study are available from the corresponding author on a request.

## Electronic supplementary material

Supplementary figures

Supplementary videos 1-3
