# Supplementary material for: Calponin-3 is critical for coordinated contractility of actin stress fibers
Source: Sci Rep. 2018 Dec 5;8:17670. doi: 10.1038/s41598-018-35948-6 (PMC6281606; doi:10.1038/s41598-018-35948-6)
Supplement: Supplementary file 1 — Supplementary figures [file 41598_2018_35948_MOESM1_ESM.pdf]

SUPPLEMENTARY DATA

Calponin-3 is critical for coordinated contractility of actin stress fibers

Katarzyna Ciuba[1], William Hawkes[2,3], Sari Tojkander[4], Konstantin Kogan[1], Ulrike Engel[5], Thomas Iskratsch[2,3], Pekka Lappalainen[1]*

[1]Insitute of Biotechnology, P.O. Box 56, 0014, University of Helsinki, Finland

[2] School of Engineering and Materials Science, Queen Mary University of London, E1 4NS London, UK

[3] Randall Centre for Cell and Molecular Biophysics, King's College London, SE1 1UL London, UK

[4] Section of Pathology, Department of Veterinary Biosciences, University of Helsinki, Helsinki, Finland.

[5] Nikon Imaging Center at Heidelberg University and Centre for Organismal Studies (COS), Heidelberg University, Im Neuenheimer Feld 267, Heidelberg, 69121, Germany.

***Corresponding author:**

Pekka Lappalainen

Email: pekka.lappalainen@helsinki.fi

Phone: +358-50-4155433

SUPPLEMENTARY FIGURES LEGENDS

Supplementary Figure 1. Cnn-3 is present in all stress fiber populations (A) 3D-SIM images of the localizations of endogenous Cnn3 and NMII in a U2OS cell. Please note that Cnn3 is present in all stress fiber categories (dorsal stress fibers are marked with red arrows, ventral stress fibers with orange arrows, and thin transverse arcs with yellow brackets). Importantly, it is also enriched in dorsal stress fibers that do not contain NMII. (B) Wide-filed immunofluorescence images of Cnn3 knockout cells demonstrating that NMIIA and α-actinin-1 (both visualized by antibodies) still localize in stress fibers of Cnn3 knockout cells. (C) 3D-SIM images demonstrating the localizations of endogenous NMIIA and α-actinin-1 as well as F-actin (visualized by fluorescent phalloidin) in Cnn3 knockout cells. Single slices of 3D-SIM images are presented. Scale bars, 10 µM.

Supplementary Figure 2. Generation and analysis of the Cnn3 knockout cell-line (A) sgRNA was designed to target the exon 1 of the CNN3 gene (labeled dark blue) with GGG PAM-sequence (labeled in red). Sanger sequencing of the first complete exon of the *CNN3* gene from wild-type (forward only), and Cnn3 knockout (forward and reverse reads) cells. (B) Next-generation sequencing (NGS) of the same region from knockout cells shows three variants. (C) Each three variant of CNN3 gene harbor a premature stop codon in the second exon, thus resulting in drastically truncated versions of the protein. Stop codons labeled as '*' – asterisk symbol.

Supplementary Figure 3. Cnn-3 si RNAknockdown. (A) siRNA SMARTPool (of 4 target sequences) was applied to obtain Cnn3 knockdown cells. (B) Depletion of Cnn3 was from siRNA cells (CNN3 siRNA) and knockout cells (CNN3 KO) was confirmed with Western Blot. anti-tubulin antibody was used a control for sample loading. (C) To visualize the Cnn3 siRNA phenotype, actin filaments (by fluorescent phalloidin staining) and focal adhesions (by anti-vinculin antibody) of mixed populations of control and Cnn3 knockdown cells were visualized. Outlines of Cnn3 knockdown cells (as detected by anti Cn3 antibody) are represented by white-dash lines. Stress fiber categories in wild-type and Cnn3 knockdown cells are marked as in Fig. 1A. Scale bars, 10 µM.

Supplementary Figure 4. Pillar displacements before and after the breakage. A comparison of the pillar positions before and after the breakage indicates that the force on pillars close to the breakage (1-3) as well as further away (4,5) drops after the event (left, red arrows indicate pillar movements above the noise level of 30nm). Right: Individual pillar traces of the pillars marked on the left show

a buildup of force on some pillars prior to the breakage event and close to the breakage point (pillars 1-3).

SUPPLEMENTAL VIDEOS

Suppl. movie 1. Control movie on stress fiber dynamics in U2OS cell, transfected with GFP actin. Images were acquired for 30 min, every 30 s. Display rate is 10 frames/second.

Suppl. movie 2. Stress fiber dynamics in Cnn3 knockdown U2OS cell, transfected with GFP actin. Images were acquired for 30 min, every 30 s. Display rate is 10 frames/second.

Suppl. movie 3. Stress fiber dynamics in Cnn3 knockout U2OS cell, transfected with GFP actin. Images were acquired for 30 min, every 30 s. Display rate is 10 frames/second.

S1

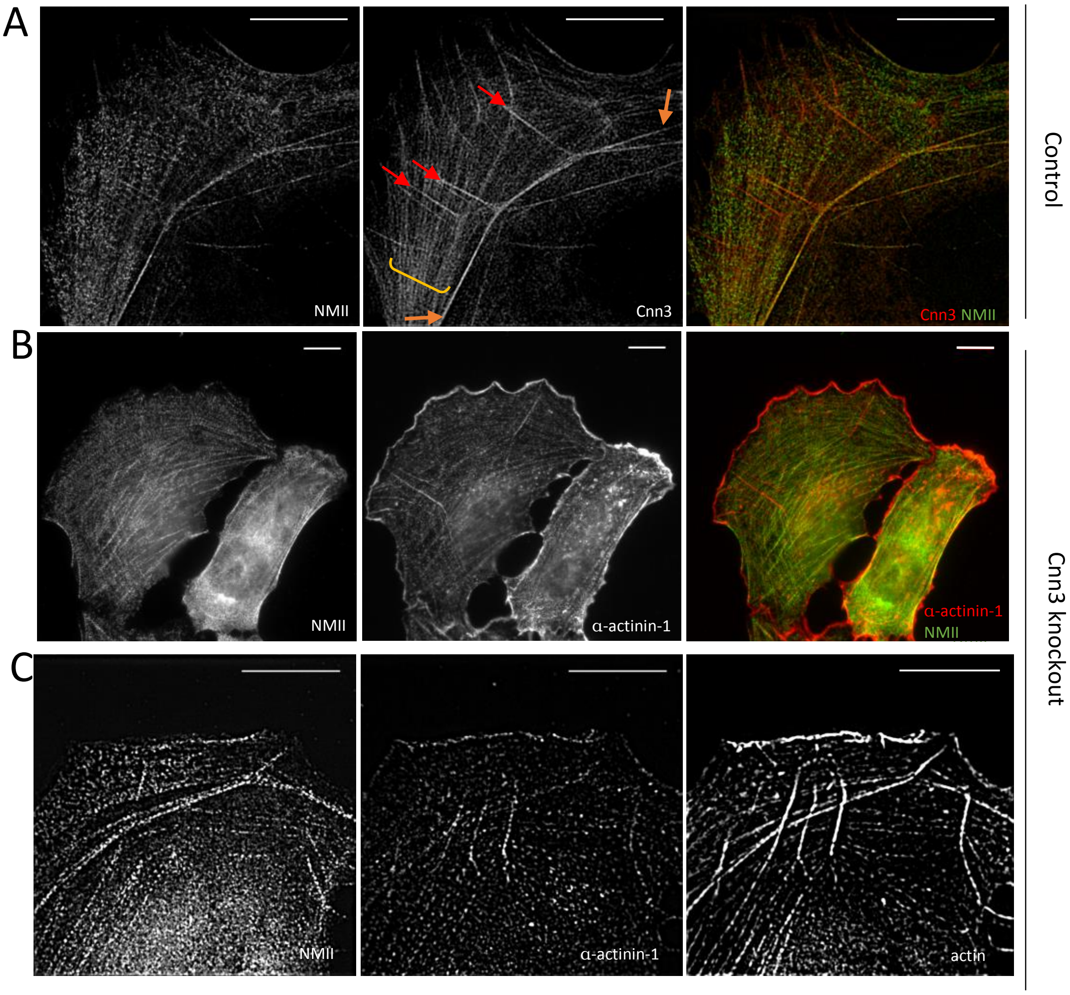
A
B
C
NMII
Cnn3
Cnn3 NMII
NMII
α-actinin-1
α-actinin-1
NMII
NMII
α-actinin-1
actin
Control
Cnn3 knockout

S2

A

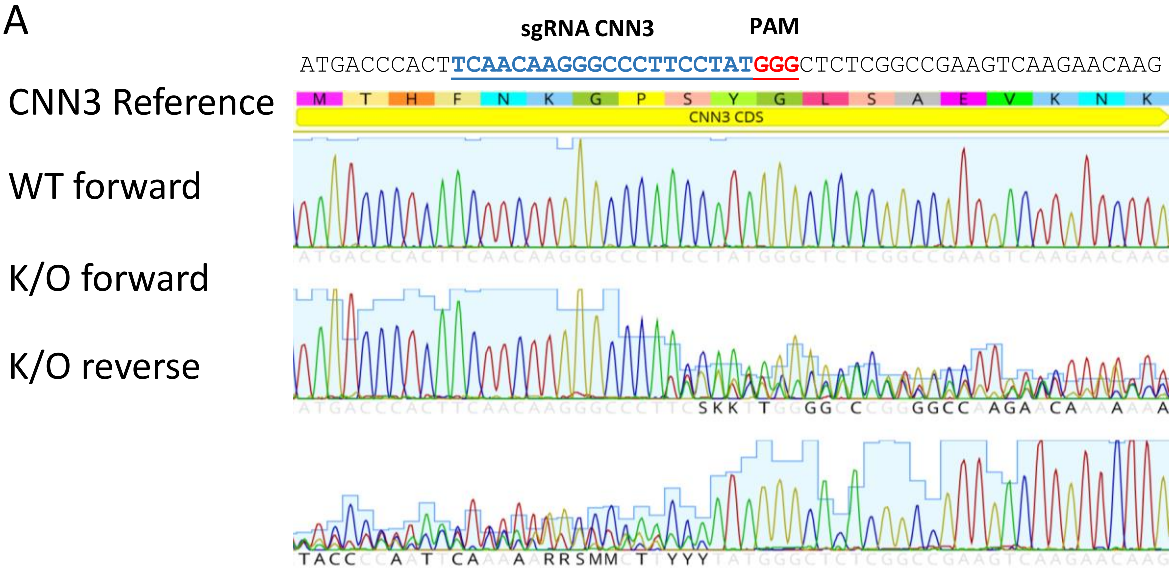

B

```
            ATGACCCACTTCAACAAGGGCCCT------ATGGGCTCTCGGCCGAAGTCAAGAACAAG
Variant #1  ATGACCCACTTCAACAAGGGCCCTTCCT--ATGGGCTCTCGGCCGAAGTCAAGAACAAG 33.8%
Variant #2  ATGACCCACTTCAACAAGGGCCCTTACTTTATGGGCTCTCGGCCGAAGTCAAGAACAAG 30.2%
Variant #3  ATGACCCACTTCAACAAGGGCCCTTCCTT-ATGGGCTCTCGGCCGAAGTCAAGAACAAG 32.5%
```

C

```
                    Exon 1                       Exon 2
Calponin 3 protein MTHFNKGPSYGLSAEVKNKIASKYDHQAEEDLRNWIEEVTGMSIGPNFQLGLKDGIILC
Variant #1         MTHFNKGPMGSRPKSRTRLLPSMIIRQKKIFAIG*KR*QA*ALAPTSSWA*RMASSSA
Variant #2         MTHFNKGPYFMGSRPKSRTRLLPSMIIRQKKIFAIG*KR*QA*ALAPTSSWA*RMASSSA
Variant #3         MTHFNKGPSLWALGRSQEQDCFQV*SSGRRRSSQLDRRGDRHEHWPQLPAGLKGWHHPLR
```

S3

A

Dharmacon ID	Target Sequence
CNN3, J-0111612-17	5'-UCUCUUACCUAGCGAACAA
CNN3, J-0111612-18	5'-GAGAUUACCAAUAUAGCGA
CNN3, J-0111612-19	5'-CAGUGAAGAAGGUCAACGA
CNN3, J-0111612-20	5'-CGGUGGACAACUCGACAAU

B

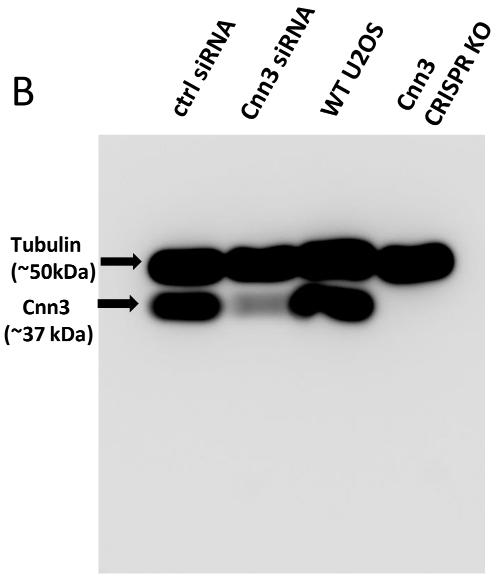

C

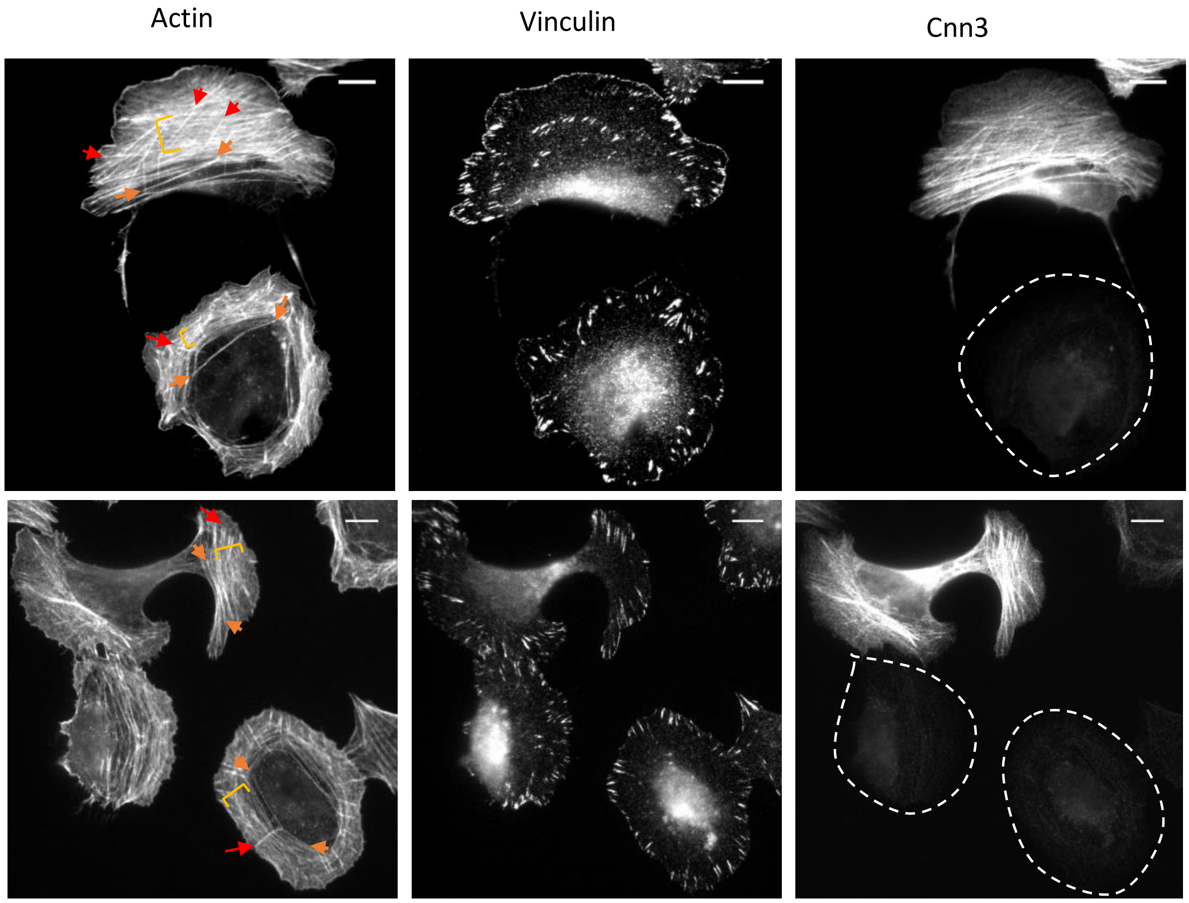

S4

A

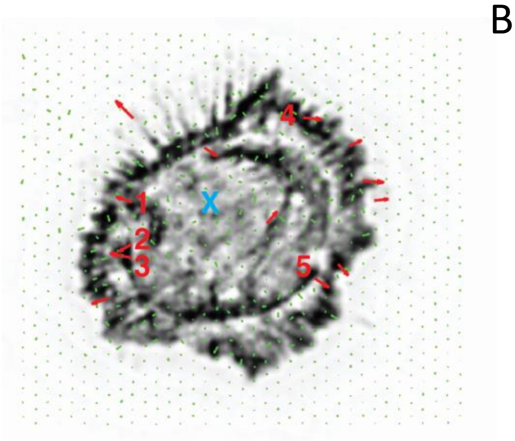

4
1
2
3
5
X

B

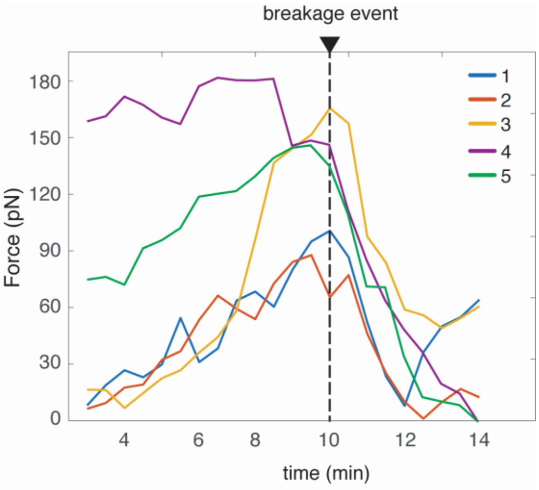

breakage event
Force (pN)
time (min)
0
30
60
90
120
150
180
4
6
8
10
12
14
1
2
3
4
5
